# Supplementary material for: Are carbon emissions trading and green financial instruments synergistic? -Comprehensive quantitative research based on content analysis
Source: PLoS One. 2024 Mar 7;19(3):e0298601. doi: 10.1371/journal.pone.0298601 (PMC10919601; doi:10.1371/journal.pone.0298601)
Supplement: S1 Data — (ZIP) [file pone.0298601.s001.zip › Data/地级市Z_F工作报告.zip!/╔╜╢1⁄2/╝├─╧╩╨/└┤╫╘╣1⁄2╓┌║┼í╛┬φ┐╦╩2╛▌═°í┐_2019.docx]

各位代表：

现在，我代表市人民政府向大会报告工作，请予审议，并请各位政协委员和其他列席人员提出意见。

一、2018年工作回顾

刚刚过去的一年，是济南跨越赶超进程中极为重要的一年。一年来，全市上下牢记习近平总书记“两个走在前列、一个全面开创”的殷切嘱托，解放思想、改革创新，雷厉风行、快干实干，全力推进“1+454”工作体系，圆满完成了市十六届人大二次会议确定的目标任务，各项工作成效显著，开创了省会经济社会发展新局面。

（一）坚持咬定发展第一要务不放松，“四个中心”建设实现历史性突破。秉持“一张好的蓝图绘到底”，注重精准施策，强化协同推进，完成了“三年有突破”的既定目标。经济综合实力整体跃升。全市生产总值达到7856.56亿元、增长7.4%，高于全省平均水平1个百分点;固定资产投资增长9.6%，高于全省5.5个百分点，增幅全省第一；一般公共预算收入完成752.8亿元、增长11.2%，税收占比达到82.3%，收入增幅和税收占比均居全省前列；进出口总额增长16.2%，高于全省8.5个百分点。主要指标增幅领跑全省，经济总量跃居全省第二位，首次进入“亚洲城市50强”，直接跻身“全球二线城市”。金融支撑作用明显增强。新引进金融机构88家，各类金融机构达到660家；完成金融业增加值831亿元、占全市GDP比重10.6%，实现税收收入144亿元，均居全省首位；全市金融机构本外币存款余额达到17060.1亿元、居全省首位，本外币贷款余额达到16059.9亿元、增长11.9%；新三板挂牌企业总数达到163家、居全省首位，融资额占全省53.8%。物流辐射效应日益凸显。国家5A级物流企业达到11家、国家级示范物流园区2家，均居全省第一；社会物流总额增长13%，从全省第五位跃升至第三位；研究出台物流专项规划，成功举办2018年中国物流企业家年会，入选国家物流枢纽承载城市，荣获“改革开放40年城市物流发展成就奖”。科技创新活力加速释放。高新技术产业产值占规模以上工业总产值比重达到56%，万人有效发明专利拥有量达到29.28件，入围“全省高新技术企业创新能力百强”企业达到19家，均居全省首位；国家级知识产权保护中心获批建设；国家科技领军人才创新创业基地（济南）正式启用；高新区生产总值突破千亿，在全国168个国家级高新区综合排名由第15位跃升至第11位，居全省第一。济南综合科技创新水平指数及增幅均居全省首位。

（二）坚持推动高质量发展不动摇，新旧动能转换呈现强劲态势。不断改造提升旧动能，大力培育发展新动能，积极创建质量强市示范城市，加快建立符合省会禀赋的现代产业体系。承接动能转换的载体加快布局拓展。450平方公里的新旧动能转换先行区直管区全面启动建设，“三桥一隧”正在强力推进，总投资1500多亿元的高端装备制造产业园等八大项目集中开工建设，储备项目超过200个；45平方公里的国际医学科学中心建设快速推进，国家健康医疗大数据北方中心、山东质子治疗中心等核心项目破土动工，山东第一医科大学将于今年9月实现招生；3.2平方公里的中央商务区“百日会战”攻坚如火如荼，“山、泉、湖、河、城”五座超高层建筑正拔地而起；西客站片区正在规划建设100平方公里的央企总部城，新东站片区正在规划建设100平方公里的省企总部城，内陆港核心区正在规划建设57平方公里的高端物流集聚区，沿经十东路正在规划建设100平方公里的齐鲁科创大走廊，多点支撑、板块联动的新旧动能转换格局初步形成。支撑动能转换的优势产业加快发展壮大。大数据与新一代信息技术、智能制造与高端装备两大产业主营业务收入均突破3000亿元，软件和信息服务业占据全省半壁江山，医疗康养、文旅产业达到千亿规模，重汽、浪潮两大企业集团营业收入突破千亿。现代金融、现代物流、人力资本产业融合发展，新一代信息技术产业集群获评全省唯一支持的支柱产业集群，大数据应用等8个产业集群入围全省首批现代优势产业集群，数量居全省首位。引领动能转换的创新成果加快落地转化。全球首个全国量子计算与测量标准化技术委员会揭牌，量子计算与量子雷达、量子芯片实现产业化；新一代神威Ｅ级原型机系统在国家超算济南中心正式启用；重汽全球首款无人驾驶电动卡车投入运营；伊莱特重工直径16米的无缝轧环机打破世界纪录；浪潮服务器市场占有率稳居全球前三，新一代服务器刷新全球最高性能纪录和最佳性价比世界纪录；齐鲁制药位居中国生物药研发50强首位，是全国唯一实现无菌注射剂对日本出口的企业。很多成长型高新技术企业，也都在各自领域科技创新方面释放出强劲活力。

（三）坚持破除瓶颈制约不止步，改革开放迈出坚实步伐。聚焦短板、抓住关键，推动改革开放向纵深发展。重点领域改革成效显著。强力推进行政效能革命，全面实施“一次办成”改革，聚焦营商环境突出问题，创造性实施“立体式”监督、“点穴式”察访、“清单式”整改、“靶向式”问责，“拿地即开工”审批模式被国务院在全国复制推广，市场主体增速由全省第16位升至第1位，总量一举突破80万户。济阳实现撤县设区，国资国企改革深入推进，医药卫生改革取得积极进展，农村产权流转交易市场体系“三台共建”模式向全国推广。我市被评为“2018中国企业营商环境十佳城市”，城市改革热度指数位居全国第五。对外开放深入推进。国际内陆港加快建设，被授予全国首个“国家智慧物流创新先行区”；新东站投入使用，石济客专、济青高铁开通运营；相继开通济南至巴黎、比利时和赫尔辛基的洲际航线，国际通达能力持续增强。全国首个泛北方区域性签证中心挂牌，中国（德国）欧洲境外企业联盟成立，境外企业联盟总数达到5个。国家侨梦苑、中德中小企业合作区等一批合作载体加快建设，国际友好城市和友好交流城市达到70个，全年入境人员增长52.1%，出入境外籍人员增长74%。双招双引成果丰硕。承办举办了儒商大会、青年企业家创新创业国际峰会、首届全国工商联主席高端峰会、中德中小企业合作交流大会、中国济南新动能国际高层次人才创新创业大赛等23项重大国际性会议活动，组织开展了美国硅谷新一代信息技术对接会、英国伦敦产融创新合作高端对话会等20余项对外招商推介活动。明确46个市直部门、投融资集团和派出机构直接参与或服务招商的职责任务，成立8个招商专班，引进市外投资形成固定资产投资增长22%，实际到账外资增长40%以上，总量跃居全省第二位。省外新签约项目投资总额位居全省第一，引进世界500强项目23个、大院大所和高端研发机构103家，泰山产业领军人才新入选数量全省第一，城市创新环境竞争力排名全国第九。

（四）坚持提升功能品质不停歇，城乡面貌焕发崭新气象。深化全国文明城市创建工作，大力实施城市提升十大行动，着力打造与现代化国际大都市相适应的生活品质之城。城市载体快速增加。建筑业增势强劲，各类建筑在建工程总面积首次突破1亿平方米，在全省遥遥领先，为增加城市发展空间、提高容纳能力提供了有力支撑。道路交通日益顺畅。轨道交通1号线提前一年建成通车，圆了泉城人民多年来期盼已久的“地铁梦”。北园大街西延高架提前开通，当年开工当年打通28条瓶颈路，150公里BRT走廊成网运行，一举退出全国十大拥堵城市行列。市容市貌更加整洁。持续开展拆违拆临、建绿透绿，全年共拆除违法建设3428万平方米，建绿透绿150.3万平方米，建成口袋公园、街头游园103处；开工建设山体公园20处，完成35座山体绿化提升项目；“1+5”特色街区综合更新赢得市民点赞，“一湖一环”景观照明和“明湖秀”项目点亮泉城；完成31条黑臭水体治理任务，华山人水和谐生态治理模式在全国复制推广。生态环境明显改善。坚决抓好中央环保督察反馈问题整改，深入实施大气污染防治十大措施，空气质量良好以上天数达到192天、同比增加12天，在169个城市排名中退出后20，蓝天白云、繁星闪烁渐成常态。文明程度不断提升。我市首部精神文明建设地方法规即将颁布实施，2个社区成功上榜全国100个优秀社区。创新路口管理、建设“公交都市”成效明显，“车让行人”“排队上下车”成为泉城一道靓丽风景线，荣获2018中国领军智慧城市称号，人民群众自豪感、荣誉感、归属感大大增强。

（五）坚持以人民为中心的发展思想不偏移，民生社会事业取得显著成效。始终以造福人民为最大政绩，注重把工作做到老百姓最惦念和揪心的地方，全年办理市人大代表建议276件、政协提案558件，全面完成18件民生实事，民生社会事业投入占市级财政支出比重达到76.6%。乡村振兴扎实推进。完成乡村振兴“1+5”规划编制，启动乡村振兴齐鲁样板百村示范行动，1县5镇50个村入选全省乡村振兴“十百千”工程示范创建名单，三涧溪村在打造乡村振兴齐鲁样板中走在全国前列。脱贫任务基本完成。全市916个贫困村全部摘帽退出，17.56万现行市定标准贫困人口实现脱贫。黄河滩区脱贫迁建加快推进，南部山区易地扶贫搬迁顺利完成，与临沂、湖南湘西州、重庆武隆区扶贫协作取得积极进展。富民增收步伐加快。大力推动创业带动就业，发放创业担保贷款7.8亿元，新增城镇就业18.95万人，农村劳动力转移就业5.2万人，城镇登记失业率2.06%、低于控制目标1.44个百分点，城镇居民人均可支配收入增长7.5%，农村居民人均可支配收入增长8.0%。社会保障拓面提质。社会保险参保人数创历史新高，被征地农民社保资金落实率达到90%以上，实施职工医保 “二次报销”政策，连续14年提高企业退休人员养老待遇，居民基本养老保险基础养老金实现“八连涨”，国家居家和社区养老服务改革试点深入推进，新增养老服务设施218处。城市低保标准提高到每人每月616元，农村低保标准提高到每人每年4928元。公立医院综合改革成果不断扩大，出院患者次均住院费用持续下降，首批入选全省医养结合示范市。生活条件明显改善。全市棚改安置房开工71351套，其中省级棚改开工46673套，提前超额完成省定任务，备受瞩目的经十一路棚户区改造项目提前竣工入住，创造了“济南速度”。整治改造老旧住宅小区761.5万平方米，既有住宅加装电梯竣工186部、占全省80%以上，彰显了“济南温度”。新建一批室内农贸市场、放心早餐店、社区肉菜店等便民服务设施。完成农村危房改造3254户、无害化卫生厕所改造13.95万户、村庄街巷硬化209个。广大人民群众生活更加便利、更加舒心。文教事业健康发展。出台中心城区基础教育设施三年建设规划，累计竣工中小学幼儿园151所，首次确立幼儿园生均公用经费制度，普惠幼儿园占比达到85.7%，开工中小学幼儿园集中就餐场所129处，实现主城区学校学生就餐和课后延时服务全覆盖，较好解决了家长“孩子上学、就餐管护”的后顾之忧。建设体育活动点3347个，市民健身更加方便。旅游消费总额突破1100亿元，增长13.3%。华阳宫古建筑群修缮工程完成并向社会开放，“泉·城文化景观”列入《中国世界文化遗产预备名单》，章丘焦家新石器时代遗址荣获全国十大考古新发现。成功举办第五届中国非遗博览会、第七届山东文博会、第二届法国尼斯国际体育嘉年华、第六届冬季畅游泉水国际邀请赛，扎实开展国家文化消费试点城市暨文化惠民消费季活动，基层综合性文化服务中心覆盖率达95%以上，让广大群众在家门口享受到了丰富多彩的“文化大餐”。社会大局和谐稳定。打造党风政风行风正风肃纪民主评议与12345市民服务热线、电视问政、电台问政良性互动的“1+3”监督格局，被作为全省深化改革的基层创新做法进行总结推广。12345热线标准化工作引领全国，电视问政品牌效应深入人心，成为推动解决问题、深化作风建设的重要抓手。平安济南建设深入推进，实现273天街面“两抢”零发案，扫黑除恶专项斗争取得阶段性成果，打掉涉黑涉恶组织团伙165个，抓获涉黑涉恶违法犯罪团伙成员1125名。安全生产形势保持总体稳定，较大以上事故实现大幅下降。食品监测合格率达到97%以上，药品监测合格率达到100%，确保了泉城人民“舌尖上的安全”。双拥共建、民族宗教、人民防空、红十字会、气象地震、应急防灾、妇女儿童、青少年、老龄、残疾人、社会科学、史志档案等工作都取得了新的进步。

过去的一年，也是原莱芜市经济社会发展发生深刻变化、取得显著成效的一年。经济发展量质齐升，全市生产总值突破1000亿元、增长7.2%；实现公共财政预算收入62.6亿元、增长11.7%。支柱产业加快壮大，精品钢、汽车零部件、高端装备制造等重点产业集群实现主营业务收入1860亿元、纳税36亿元，分别增长12%和18%。改革开放持续深化，供给侧结构性改革、投融资体制改革、“放管服”改革取得积极进展。城乡融合发展步伐明显加快，扎实推进环境卫生整治、硬件设施提升、行业卫生管理等工作，集中解决了一批城乡管理的突出问题。民生保障实现新提升，创新实施市民大病补偿制度，全市二级以上医院全部纳入全国异地就医联网结算平台，人民群众在改革发展中得到了更多实惠。

各位代表！一年来，面对复杂多变、风险挑战明显增多的国内外环境，全市上下在市委的坚强领导下，以“功成不必在我、建功必须有我”的担当谋发展，以改革开放创新的精神破难题，以“不靠刮风靠作风”的劲头抓落实，干出了众志成城、昂扬向上的新气象，干出了实实在在、收获满满的新业绩，干出了生机勃勃、充满希望的新天地，济南莱芜区划调整获得国务院批复，在省委2018年度履行全面从严治党责任评议和抓基层党建述职评议排名中均居全省第一，我们实至名归地荣获全国“新时代新担当新作为先进典型”称号！这些成绩的取得来之不易，这是以习近平同志为核心的党中央坚强领导、亲切关怀的结果，是省委省政府科学决策、正确领导的结果，是几大班子精诚团结、通力协作的结果，是广大党员干部顽强拼搏、扎实苦干的结果，是社会各界和人民群众积极参与、鼎力支持的结果。在此，我代表市人民政府，向全市人民，向给予政府工作大力支持的人大代表、政协委员，向各民主党派、工商联、无党派人士、各人民团体，向中央和省在济单位和各类驻济机构，向驻济人民解放军、武警官兵，向参与城市建设的劳动者，向所有关心支持济南发展的港澳台同胞、海外侨胞和国际友人，表示崇高敬意和衷心感谢！

在看到成绩的同时，我们也清醒地认识到存在的问题和不足，主要表现为：省会优势尚未得到充分发挥，辐射力、带动力、影响力还不够强；经济发展的质量效益还不够高，高端制造业的优势还未形成，外向型经济、县域经济、民营经济仍是短板；创新驱动能力有待加强，新经济新动能培育力度还不够大；开放水平与先进城市还有较大差距，国际化程度有待提高；资源环境约束趋紧，治污减霾、生态环境保护任务还十分繁重；民生保障存在一些薄弱环节，涉及群众切身利益的住房上学交通看病养老等方面，还有许多不尽人意的地方；城乡二元结构矛盾突出，城乡居民收入差距仍然较大；许多领域不稳定不确定因素相互交织，防范风险的压力仍然较大；政府职能转变还不够到位，资源要素市场化配置程度不高；部分政府工作人员综合素质还不能适应新的要求，“四风”问题仍然存在，等等。对此，我们一定直面问题、敢于担当，全力以赴加以解决。

二、2019年经济社会发展

主要目标和总体要求

2019年是新中国成立70周年，是全面建成小康社会的关键之年，也是省会抢抓区划调整千载难逢历史机遇，奋力向着做大做强省会城市目标迈进的决定性一年。2019年全市经济社会发展的总体要求是：以习近平新时代中国特色社会主义思想为指导，深入贯彻党的十九大精神和习近平总书记视察山东重要讲话、重要指示批示精神，坚持稳中求进工作总基调，坚持新发展理念，坚持推进高质量发展，坚持以供给侧结构性改革为主线，坚持深化市场化改革、扩大高水平开放，紧紧围绕贯彻落实市委确定的“1+474”工作体系，咬定“大强美富通”现代化国际大都市目标不动摇，坚定不移打造区域性经济中心、金融中心、物流中心、科创中心，集中精力打好新旧动能转换、城市治理、双招双引、营商环境、乡村振兴、生态环保和交通枢纽建设攻坚战，坚决守住党风廉政、安全稳定、社会民生和风险防控底线，加快把济南这个山东龙头扬起来，当好新时代全省走在前列的排头兵！

综合考虑国内外经济形势和省会发展实际，今年经济社会发展的主要预期目标是：全市生产总值增长7.5%左右；一般公共预算收入增长9.5%左右；固定资产投资增长11%左右；社会消费品零售总额增长11%左右；城镇居民人均可支配收入增长7.5%以上，农村居民人均可支配收入增长8.0%左右；城镇登记失业率控制在3.5%以内；居民消费价格涨幅控制在3%以内；单位生产总值能耗下降率及主要污染物减排量达到省控目标。这些目标的确定，体现了落实走在前列的目标定位，贯彻了高质量发展的要求，统筹考虑了我市发展各方面的因素。确定这样的目标，既有利于鼓励广大干部积极作为，又有利于稳定社会预期、提振各方士气，凝聚方方面面的力量更好地自我加压、争先进位。

做好今年的政府工作，必须牢牢抓住省会强势崛起的重大历史机遇。世界正面临百年未有之大变局，我国发展仍处于并将长期处于重要战略机遇期，挑战和机遇同生并存。当前，我们面临着中美贸易摩擦影响、防控风险难度提高、经济下行压力加大等困难，但从我市看，历史的原因我市的经济外向度暂时相对较低，受中美贸易摩擦影响较小，这也同时造就了我们发展省会经济的空间大、机遇多；由于我们前期的主动作为，政府债务水平较低，企业经济风险点也基本化解，防范风险压力相对减轻，这为我们创造了轻装上阵的发展机遇；同时，国家、省对济南支持力度不断加大，做大做强省会城市、发挥省会城市龙头作用的政策效应落地有力。应该讲，省会正处于弯道超车的绝佳时期。机遇不会一而再地眷顾我们，我们一定要善于变危为机，趋利避害、扬长避短，抓住机遇、奋发作为，加快推动省会跨越赶超。必须用心把握稳中求进的工作总基调。“稳”，就是要进一步强化大局意识，守好底线、争取主动，保持好政策、工作的连续性和稳定性，坚定企业发展信心，引导好社会预期。“进”，就是要始终保持永不懈怠的精神状态和一往无前的奋斗姿态，咬定发展第一要务不动摇，咬定以经济建设为中心不动摇，咬定招商引资招才引智不动摇，咬定项目建设不动摇，保持定力、精准发力、持续用力，决不能一味强调“稳”而被动等待、消极作为，一定要瞪起眼睛、鼓足干劲、大干快上！必须始终坚持高质量发展这一根本要求。坚持高质量发展，核心是由单纯地追求经济发展的高速度，转向追求更有效率、更可持续的增长，但绝不是放弃对发展速度的要求。迄今为止，GDP仍然是衡量一个国家地区总体实力的主要指标，在坚持高质量发展的前提下，没有总量的“大”，就谈不上“强”，更谈不上“美”“富”“通”。习近平总书记要求山东、济南走在前列，就是要求我们在确保高质量发展的基础上实现快速增长。我们一定要准确把握“高”与“快”的关系，只要符合五大发展理念的GDP，有多快要多快、有多高要多高。必须紧紧扭住实施新旧动能转换重大工程这个“牛鼻子”。新旧动能转换既是实现省会高质量发展的战略支点，也是赢得区域竞争优势的强力抓手。新旧动能转换没有捷径可走，一条是培育壮大现有企业，加大企业技术改造力度，加快传统产业转型升级步伐，巩固和拓展产品市场占有率。再一条是招商引资招才引智，瞄准城市发展所需要的产业布局，全力以赴招商引资招才引智，通过招项目引团队引人才，通过招人才引团队引项目。招商引资招才引智是最快最直接见效最明显的产业结构调整，也是最好的动能转换。还有一条是着眼长远大力实施创新驱动发展战略，立足省会优势，超前谋划布局实施一批大科学计划和大科学工程，搭建一批科技创新转化平台，转化培育一批科技引领型企业，为未来发展打下坚实基础。必须正确处理令行禁止和探索创新的辩证关系。我们开展任何工作，都必须以贯彻党中央要求为前提，立场坚定、旗帜鲜明，在贯彻落实中央决策部署、省委工作要求上不打折扣、不做选择、不搞变通，确保令行禁止。但是，我们决不能以此为借口，机械执行、照搬照抄、搞上下一般粗，必须在吃透上级精神的基础上，紧密结合济南实际，创造性地抓好贯彻落实。只要有利于强化党的领导，有利于落实新发展理念，有利于维护人民群众根本利益，就要不计个人得失，敢于闯、敢于试、敢于改，推动各项工作不断实现新的突破。

三、强力推动全域一体融合发展

济南区划调整是党中央、国务院和省委省政府，立足全省发展大局，充分发挥区域比较优势，深刻把握区域经济发展规律基础上作出的重大科学决策，这是最大的新动能，对于做大做强省会具有里程碑意义，必将推动省会建设进入一个新的发展阶段。我们一定要牢牢抓住这一重大历史机遇，以对党负责、对人民负责、对历史负责的态度，精准把握融合发展的工作重点、时序步骤和力度节奏，既做“加法”，更做“乘法”，切实做好一体布局、优势互补、融合发展这篇大文章，用忠诚和担当向党中央、向省委省政府、向全体市民交上一份合格答卷。

（一）高质量推进规划融合。围绕架构起世界级城市的大框架、大格局，坚持世界眼光、国际标准、省会特色，聘请国际一流规划团队，对全域空间格局、功能区划、产业布局等进行一体规划，加快作出系统、全面的整体安排。立足发挥空间布局整体优势，把莱芜区和钢城区纳入济南新旧动能转换先行区，作为先行区的“南翼”，编制专门规划，实现产业对接、规划衔接、一体发展，加快打造新旧动能转换的核心引领优势。

（二）高质量推进产业融合。着眼构筑符合省会禀赋的现代产业体系，大力强化产业一体布局，统筹大数据与新一代信息技术、智能制造与高端装备等十大千亿级产业，与莱芜区钢城区精品钢等十五个产业集群，打造一批千亿级产业集群，形成更加合理的国土开发格局和产业发展布局。坚持强强联合、优势整合，将莱芜区钢城区作为“智造济南”的重要节点、“文旅济南”的重要区域、“康养济南”的重要载体、“全域济南”的重要支撑，着力进行“四个一体化”打造。充分发挥莱芜区钢城区生态、交通优势，深度利用济南科技教育优势资源，规划建设高水平的科学园区和国家级精品钢制造基地，加快构筑科技与创业融合的创新创造创富高地。

（三）高质量推进基础设施融合。立足建设国家重要交通枢纽，编制面向未来的交通发展战略规划，强化国际交通枢纽地位。在国际国内交通大格局中，超前谋划建设一批具有标志性的重大工程，强力推进济郑、济滨、济济（宁）、莱临（沂）等重大交通设施建设，构建“米”字型高铁网。把“济莱城铁”及早提上建设日程，争取早立项、早开工、早投用，推动省会都市圈全域轨道交通网加速形成。加快“三环十二射”高速公路、轨道交通以及顺河高架南延快速路、旅游路东延等城市重点交通设施建设，确保轨道交通R3线今年国庆节通车。推进大型交通节点和交通工具之间的有机衔接，推动全域公交一体化。加快济南国际机场扩建工程建设进度，高标准打造4F级机场，推动实施小清河复航工程，打造省会通江达海的“黄金水道”。

（四）高质量推进公共服务融合。聚焦打造宜居宜业宜游宜学的省会城市，优化住房、养老、医疗、休闲、购物等配套设施规划布局。按照标准化、便捷化、普惠性的原则，加大投入保障力度，加强各类公共服务有效供给。按照省会的标准，加快提升莱芜区钢城区教育、文化、医疗、旅游等公共服务设施建设水平和群众生活质量，确保两区与济南原有区“等高对接”，最大程度地释放区划调整的改革红利。

四、2019年政府主要工作任务

当前，省会发展已经站在了新的历史起点上。新起点要创出无愧于时代的新业绩、赢得决胜于未来的新优势。我们要乘势而上、奋发作为，统筹兼顾、突出重点，推动各项工作卓有成效地开展，为加快跨入“万亿俱乐部”、建设国家中心城市奠定坚实基础。

（一）聚焦推进“四个中心”建设，塑造省会辐射带动新优势。坚持实体经济、科技创新、现代金融、人力资本协同发展，制定实施“四个中心”建设行动计划和专项行动方案，推进“四个中心”建设提速增效。

做大做强区域性经济中心。大力发展总部经济。加快央企总部城、省企总部城规划建设步伐，用足用好支持总部经济发展的十大政策，组织开展各种形式的精准招商推介会，主动对接京津冀和雄安新区建设，主动承接北京非首都功能，积极引进上市公司和大型企业总部、区域总部、研发中心以及金融系统内服务类机构和后台服务机构，加快形成我国北方高端产业、科技、人才、现代服务业集聚地，打造央企、跨国公司区域总部基地。大力发展县域经济。强化“项目为王”理念，扎实开展“项目落地建设竞赛年”活动，统筹推进重大项目建设，确保签约项目早落地、落地项目早开工、开工项目早达产。继续强力抓好标准厂房建设，各区县分别建成面积不少于50万平方米的标准厂房，让更多企业实现拎包入住，打造小微企业专业化园区群。有针对性地制定扶持县域经济加快发展的政策措施，让县域经济在省会发展中担重任、挑大梁。大力发展消费经济。把促进消费、扩大内需作为拉动经济增长的主动力之一，积极培育教育、育幼、养老、医疗，特别是文化旅游等消费新热点，扎实开展国家文化消费试点城市暨文化惠民消费季活动，申报全国高品位步行街创建试点，大力发展全域旅游，建设国际旅游目的地。研究制定促进工业产品消费的意见，加大政府采购力度，促进工业经济集群发展和供需有效对接。出台促进消费升级的实施意见，抓好省会商圈提升，创建国家跨境电子商务综合试验区，拓宽国际中高端消费品购买渠道，打造“全国放心消费城市”。

做大做强区域性金融中心。围绕增强金融集聚能力，加快CBD金融城项目建设步伐，完善汉峪金谷配套设施，推进山东新金融产业园二期工程，重点引进财务公司、基金公司、外资银行等金融业态和机构，打造全牌照、全业态金融机构集群，力争金融业增加值达到940亿元、增长8.5%左右。围绕增强金融服务能力，实施产融融合工程，强化政金企合作，引进培育金融中介服务机构，组建大型政策性融资担保集团，通过拨贷联动、风险补偿等方式，引导更多社会资本投入实体经济。加大直接融资力度，支持企业有选择地开展短期融资券、中期票据、中小企业集合票据等债务工具融资，提升创新型债券利用水平。开展企业上市突破年活动，新增境内外上市挂牌企业15家以上，打造资本市场“济南军团”。围绕增强金融抗风险能力，持续优化金融生态环境，设立中小微企业贷款风险补偿资金池，搭建金融风险监测预警平台，加强对小额贷款公司、融资租赁公司、权益类交易场所等地方金融组织监管，确保不发生区域性系统性金融风险。

做大做强区域性物流中心。以建设“路港、空港、保税港、信息港”四港合一的国际内陆港为抓手，深入推进国家供应链体系建设综合试点。加快建设高端物流集聚区，开工建设国际陆港大厦，加快董家铁路货运枢纽建设，设立铁路一类口岸，构建多式联运综合枢纽，在“齐鲁号”欧亚班列运营中确立优势主导地位。突出抓好传化公路港、零点国际物流园、菜鸟物流等重大物流项目，加快保税仓储中心、大数据信息服务等综合配套体系建设。大力发展无车船承运、多式联运、城市共同配送、冷链运输等高端物流及智慧物流，打造区域集散转运中心，社会物流总额增长11%，规模以上物流企业营业收入增长9%，新增规模以上物流企业50家。

做大做强区域性科创中心。发挥企业创新主体作用。建设以企业为主体的自主创新体系，实施高新技术企业梯次成长计划，发挥新旧动能转换基金作用，加大服务支持和资源倾斜力度，高新技术企业总数达到1850家以上，高新技术产业产值增长10%以上。实施企业研发能力提升工程，支持大中型工业企业和规模以上高新技术企业建设研发平台，鼓励企业并购或设立海外研发机构，全社会研发经费增长15%。充分发挥省会高校和科研机构集聚的优势，建设以高校科研单位为引领的基础和应用基础研究体系，鼓励面向省会未来发展和十大千亿级产业的高校科研单位、创新团队、企业及领军人才等联合创新，构建产学研相结合的高效创新转化机制。积极创建国家制造业创新中心，加快高新区争创世界一流高科技园区的步伐。发挥各类科创平台保障作用。积极对接省级100项重大技改项目和20项省级大科学计划、大科学工程及30个科学实验室，提升重塑齐鲁软件园开发平台，着力抓好山东产业技术研究院承建工作和山东工业技术研究院提升工程，推进中科院协同创新中心、深圳先进技术研究院落地，积极对接省直有关部门承建山东大生命科学工程产业技术研究院，以浪潮集团牵头组建现代信息产业技术研究院，以山钢集团牵头组建精品钢产业技术研究院，以济南量子技术团队牵头组建量子产业技术研究院，以国家超级计算济南中心牵头组建超算及人工智能产业技术研究院，以重汽集团牵头组建氢能源及燃料电池应用技术研究院，以天岳公司会同山东大学牵头组建第三代半导体产业技术研究院，以韩都衣舍等优势电商企业牵头创新电子商务孵化应用平台，打造“政产学研金服用”创新创业综合体。发挥成果转化服务体系助推作用。推进科技人才资本化，建立科技金融融合新机制。出台以校地合作项目为核心的扶持办法，促进科研成果在省会孵化转化落地。深入推进国家科技成果转移转化示范区建设，建设中国（济南）知识产权保护中心，鼓励发展创新工场、虚拟创新社区等新型孵化器，大力推动大众创业、万众创新。

（二）聚焦加快新旧动能转换，塑造省会高质量发展新优势。把实施新旧动能转换重大工程作为推动省会高质量发展的总牵引，以更大的力度推动存量变革、增量崛起，切实增强省会经济创新力和竞争力。

推动先进制造业集群发展。济南正处于工业化发展后期，而不是后工业化时期，亟需布局和完善现代产业体系。实施工业强市战略，落实促进先进制造业和数字经济发展的政策措施，加快出台智能制造与高端装备、大数据与新一代信息技术、量子科技、生物医药等产业的细分行业产业发展规划和行动计划，围绕高端装备、先进材料、新能源汽车、精品钢材等先进制造业产业，构建优势制造业产业集群。实施新一轮企业技术改造，大力实施“现代优势产业集群+人工智能”“人工智能+”工程。积极争取工业互联网解析节点落地济南，力争上云企业总数突破1万家，努力打造“济南智造”品牌。

推动现代服务业快速发展。推进软件名城提档升级，全力打造国际会展名城，加快引进国际法律、会计、咨询等高端服务机构，大力发展研发设计、检验检测、人力资本等生产性服务业，促进生产性服务业向专业化和价值链高端延伸。制定出台关于创新发展生活性服务业的意见，大力发展智慧服务、体验服务、定制服务、共享服务、绿色服务、跨境服务等新兴服务形态，促进生活性服务业向精细化高品质转变，形成布局合理、功能完备、优质高效的生活服务体系。

推动新旧动能转换先行区引领发展。突出抓好产业带动，强力推进高端装备制造产业园、国际会展中心等一批重点项目建设，推动京东智能电商运营中心、中科院新经济科创园等一批项目签约落地，高标准规划建设齐鲁科学城。突出抓好基础支撑，在加快“三桥一隧”建设的基础上，再规划建设一批跨河通道，强力推进轨道交通建设，统筹水、电、气、热、通信等增源扩能、布点建站，完善市政道路等基础设施配套。突出抓好地标塑造，高标准规划建设政务服务中心、文化艺术中心、体育活动中心、核心区中央公园，加快申建黄河国家湿地公园，增强先行区的承载力、集聚力和吸引力。

（三）聚焦提高“双招双引”质效，塑造省会快速壮大新优势。坚定不移抓好“双招双引”一号工程，秉持绿色招商理念，聚焦项目高端、产业优质、发展高效，以更大的力度、更实的举措，推动“双招双引”取得更加丰硕的成果。

强化招商引资。注重产业链招商，成立产业招商专门队伍，瞄准香港金融、欧洲智能制造、日韩高新技术产业、美国创新资源等，以全球视野找准产业链上的新技术、新产品，开展专业化精准招商。注重依托国际国内有影响的中介机构、社会组织，发挥全球招商合伙人资源优势，提高招商质量。注重用好亲情纽带，突出抓好校友招商。注重发挥企业以商招商作用，引导支持优势企业实现点对点招商，形成“引进一个、建好一个、带来一批”的良好效应。

强化招才引智。深入实施泉城“5150”引才倍增计划、泉城产业领军等重点人才工程，加快建设济南“院士谷”，引进一批海内外高精尖人才团队，新增院士专家工作站40家，推进国家海外人才离岸创新创业基地建设，争创国家级人力资本产业园。开展“济智回归”行动，新建一批专家公寓、人才公寓、大学生公租房，积极吸纳高校毕业生在济就业，打造年轻人友好城市。深入实施外籍人士“家在泉城”工程，加快建设一批国际学校、国际医院、国际社区，全方位提升国际化人才服务水平。

强化督导考核。明确驻海外机构招商主责，调整驻外办事机构职能职责，把招商引资作为主业，不断壮大招商队伍。建立市级领导包挂推进重点招商项目制度，强化已签约项目的落地。建立科学合理的“双招双引”考核体系，强化考核结果运用，层层传导压力，真正让“双招双引”没有局外人、没有旁观者。

（四）聚焦深化改革扩大开放，塑造省会国际竞争新优势。深化市场化改革，扩大高水平开放，深入落实对外开放40条，把制度创新作为补齐短板、加固底板最根本的改革任务，最大限度地激发各方面创新创造活力。

创新园区体制机制。加快推进园区综合改革，以去行政化为方向，以省级以上开发区、综保区和国际医学科学中心为重点，大力推行“管委会+公司”模式，建立灵活的用人制度和薪酬体系，推动园区实现企业化管理、市场化运作、专业化服务、绩效化考核、特色化发展，让各类园区真正成为改革开放的先行区、创新发展的引领区、“双招双引”的示范区。

深化国资国企改革。健全完善国有资本投资公司、运营公司管理运营方式，推动六大投融资集团向特色化、专业化、市场化国有资本投资运营公司转变。加快国有经济结构调整和战略性重组，大力推进混合所有制改革，推出50个市属国有企业混改项目，激发国有企业内生动力。

深度融入“一带一路”国际合作。加快推进中德中小企业合作示范区、国家侨梦苑等平台建设，积极申建中国（山东）自由贸易试验区济南片区，确保新迁建综合保税区12月底前封关验收。积极参与中国—中东欧国家地方经贸合作示范区建设，推进对外合作平台载体提质升级。着眼在更大的国际国内市场空间内整合要素资源，重点围绕十大千亿级产业，找准技术、资本、人才、市场的互动点，深度融入共建“一带一路”。重点支持浪潮、重汽、齐鲁制药等骨干企业积极开展国际投资、海外研发，推动设计咨询、投融资、建营一体化企业抱团走出去，实现“海外济南”规模跨越式增长。

进一步扩大对外交往。精心承办鲁港通、中国企业500强高端峰会、青年企业家创新发展国际峰会、工商联专业分会、全国工业机器人应用技术技能大赛等重大赛会活动，进一步做大中德中小企业合作交流大会、国际泉水文化景观城市联盟会议、市长国际经济咨询委员会年会、东亚博览会、中国济南华侨华人双创大会、第十届华商企业科技创新合作交流会等特色国际品牌活动，精心举办“外交官走进泉城”系列活动，推动与外国使领馆建立创新合作机制实现新突破。

（五）聚焦提升城市治理水平，塑造省会功能品质新优势。把民生幸福作为城市发展的最高追求，深入推进城市提升十大行动，全面抓好“城市双修”、城市设计全国试点，着力推动城市更新，优化提升老城区，科学开发新城区，加快建设大气秀美、清新靓丽、古今交融、品质上乘的时尚之城。

强化市级统筹。抓好城市规划市级统筹。把规划作为引领城市发展的指挥棒，完善城市规划制度，高质量完成济南城市发展战略规划，对城市建设特别是重大基础设施建设，由市级统一进行规划，形成“市级统筹、分区实施”的工作格局。抓好土地收储市级统筹。成立市土地储备开发主体，强化全市土地资源统一收储、配置、监管，实现土地资源配置“一个渠道进水、一个池子蓄水、一个龙头放水”。按照“总量控制、存量优化、质量提高”的原则，强化土地全生命周期共同监管，探索建立“标准地”制度，加强绩效评价和退出监管，以强度换空间、以空间换品质。抓好财政收支市级统筹。完善预算执行管理机制，全面实施预算绩效管理改革，切实提高市级财政统筹能力，增强资金使用的精准性，集中财力办大事。抓好产业布局市级统筹。根据各区县自身禀赋、产业基础和全市产业总体规划，由市级统筹产业布局，推动每个区县形成自身的主导产业，实现错位发展、特色发展、协调发展，形成“各唱各的拿手戏、各打各的优势牌”的良好局面。

强化分区治理。对历史传承区，坚持“留、改、拆”并举，坚决拆除违章建筑，采取保守治理措施，进行局部修整和功能置换。突出抓好“泉城大客厅”建设，做好泉城特色风貌带规划以及千佛山周边、“一湖一环”周边、历史文化街区城市设计，加强历史建筑保护，大力疏解中心城区非核心功能，集中打造集山、泉、湖、河、城为一体的“城市绿心”。对“三旧”改造区，进行精心设计重塑，解决老城区环境品质下降、空间秩序混乱问题。按照旧村改造市级统筹的原则，妥善处理历史遗留问题，对具有保护价值的传统建筑实施产权回购、有机更新，将符合控制性详细规划的更新项目委托区县实施。加大征收拆迁力度，高标准推进36个棚户区改造项目，开工建设棚改安置房4.4万套，实施358个老旧小区、721万平方米改造整治，既有住宅加装电梯200部。把开展拆违拆临的范围扩大到城乡全域，坚持一把尺子量到底，确保拆除3000万平方米以上的违法违规建设。对新开发区域，研究出台建筑设计导则，完善各类建筑标准体系，保证城市环境、文脉、建筑美学高度契合，确保重要建筑物、构筑物建成“精品工程”，塑造城市新形象。健全城市建设长效机制，突出抓好地下管网建设管理改革，构建现代基础设施体系。大力开展“无违建”街镇创建行动，力争无违建街镇达到50%以上。持续实施建绿透绿，对以经十路为重点的100条主次干道和城市出入口进行改造提升，新建一批街头公园、文化雕塑、景观小品，努力实现一路一景、三季有花、四季常绿、扮靓泉城。

强化精细化管理。既要善于运用现代科技手段实现智能化管理，又要通过绣花般的细心、耐心、巧心提高精细化管理水平，绣出城市的品质品牌。推动城市管理创新转型升级，实行以社区为管理单元、辅以网格化管理的基层管理体制，长效开展社区微治理，有效解决老旧小区乱停车、楼道杂物堆积、生命通道占用、非法张贴小广告等城市顽疾。大力推行路长制，联动开展交通秩序、城市治安、市政设施、城市家具、广告店招、街面秩序、空间立面、环境卫生、园林绿化等综合治理。实施城市亮化提升工程，出台城市灯光管理办法，逐步实现城市照明设施及光亮设施智能管控，建设一座具有独特韵味的“不夜城”。

强化文明城市建设。深化巩固文明城市创建成果，深入实施文明交通行动，大力实施智慧应用“七通”工程，在智慧交通、智慧城管、智慧医疗、智慧警务等领域实施一批智慧民生示范项目，让市民充分享受现代文明成果。充分发挥“泉城义工”“旅游啄木鸟”等志愿服务品牌示范作用，推动文明城市创建向县镇村拓展延伸。扎实开展“书香泉城”创建活动，实施“泉城阅读工程”，建设一批24小时泉城书房，打造一座充满书香味的儒雅之城。

（六）聚焦优化营商环境，塑造省会市场活力新优势。坚持问题导向、需求导向和目标导向，从企业最不满意的地方入手，从群众最期盼的事情做起，锲而不舍推动营商环境持续好转。

大力开展行政效能革命。强力推进“一次办成”改革，在抓好“一次办”的基础上，加大数据资源整合力度，加快实现“一网办”“指尖办”“马上办”。加大对中介机构的扶持规范力度，加快中介机构“进厅上网”。学习借鉴国务院推广的优化营商环境典型做法，切实做到“企业有需要、部门去报到”，充分激发各类市场主体发展活力，确保全年市场主体总量突破110万户。

加快推进社会诚信体系建设。健全企业法人、社会组织、机关事业单位和自然人四个信用数据库，打造横向联通、纵向连接的智能化信用信息共享平台。梳理涉企承诺事项，严格落实对企承诺，将各单位的守信践诺情况纳入政务诚信记录，依法依规处理“新官不理旧账”的问题。制定推出社会信用管理办法，拓宽加大对失信被执行人联合惩戒的范围和力度，努力打造“信用泉城”。

激发民营经济发展活力。全面落实扶持民营经济发展的各项政策，清理废除阻碍统一市场和公平竞争的规定做法，加快培育一批瞪羚、独角兽和平台生态型龙头企业。建立民营企业风险缓释服务机构，用足用好新旧动能转换企业纾困基金。积极引导城商行、农商行等地方金融机构回归本源支持企业发展。聚焦民营企业融资难融资贵等痛点难点焦点问题，充分发挥“1+3”平台作用，向损害营商环境的违纪违法行为亮剑，推进“金牌环境”提升。制定政商交往正面清单和负面清单，构建“亲”“清”新型政商关系。像保护眼睛一样保护民营经济，像尊重科学家一样尊重企业家，召开服务支持民营经济发展大会，表扬激励一批作出突出贡献的民营企业家，大力弘扬企业家精神和新时代儒商精神，切实让企业领跑城市、让市场活跃城市、让政府温暖城市。

（七）聚焦实施乡村振兴战略，塑造省会城乡融合发展新优势。实施以村庄为单位的乡村振兴工作机制，整合涉农资金，以村为单位统筹安排资金使用方向，逐村摸情况、出思路、定举措，推动省会在打造乡村振兴齐鲁样板中走在前列。

突出抓好规划引领。健全市级主导、区县主体、街镇实施、部门配合、群众参与的规划工作机制，完善实施乡村振兴战略规划；围绕推进新型城镇化，按示范引领、特色保护、改造提升、搬迁撤并等类别，对村庄进行分类管理。搞好单个村庄规划设计，提升街镇建设规划，确保所有保留的村居“一村一本规划”，形成与省会风格风貌相匹配的农村发展新格局，让群众望得见山、看得见水、记得住乡愁。

突出抓好改革驱动。坚持多予少取放活,统筹推进农村土地制度改革，全面完成集体产权制度改革，积极探索承包地“三权分置”有效实现形式，稳慎推进宅基地“三权分置”改革试点。探索推进集体经营性建设用地入市改革，加快壮大农村集体经济。开展资源性资产确权登记颁证工作，深化农村产权流转交易市场体系“三台共建”机制，探索农民专业合作、供销合作、信用合作“三位一体”的农村新型合作体系，不断释放资源要素潜力、激活农村发展动力。

突出抓好产业振兴。坚持质量兴农、品牌强农、科技助农，加快农业新旧动能转换，健全现代农业经营体系，推进一二三产业融合发展。着力打造一批乡村旅游示范镇和精品线路，推动乡村旅游规模化精品化发展。办好中国玫瑰文化艺术节、中国草莓文化旅游节，壮大平阴玫瑰、章丘大葱等“十朵金花”，提升“三辣一麻”“三黑一花”品牌价值，打造都市精致农业特色产业集群。发展壮大农产品加工示范园区、示范企业，以点带面，拉长种养产业链条，补齐农产品深加工短板，让工业牵着农业的手、城市带着乡村走。

突出抓好典型示范。大力实施农村人居环境整治三年行动计划，深入推进农村“七改工程”，推动优质教育、文化、医疗等公共服务向农村延伸，建设一批特色小镇，推动新时代文明实践中心覆盖全市66%的村居，打造乡村振兴齐鲁样板村105个，同步带动建设100个示范村，以点带面、步步为营，带动省会逐步实现农业强、农村美、农民富。

突出抓好精准帮扶。推进脱贫攻坚与乡村振兴相衔接、开发式扶贫与保障性扶贫相结合，确保贫困人口“两不愁、三保障”。建立脱贫成效巩固提升监测机制，对已脱贫贫困村、贫困人口“回头看”“回头帮”，积极推进贫困村、贫困户人居环境改善工作。高质量高标准完成黄河滩区迁建年度任务，提升与临沂、湖南湘西州、重庆武隆区的帮扶合作水平，抓好援藏援疆工作。

（八）聚焦加强生态环保建设，塑造省会可持续发展新优势。牢固树立“绿水青山就是金山银山”的理念，深入开展“四减四增”三年行动，狠抓中央、省生态环境保护督察反馈意见整改落实工作，坚决打好污染防治攻坚战，建设“天蓝地绿、山青水秀”的美丽家园。

坚持不懈治霾。深入实施蓝天保卫战三年作战计划，强化工业企业和柴油车污染治理。完善重污染天气应急预案和重点时段减排要求，落实好“蓝绿名单”制度，依法分类处置“散乱污”企业。稳妥推进主城区“退二进三”或“退二优二”，着力加快“外热入济”。统筹推进清洁取暖工作，积极推进煤改电、煤改气工程，切实抓好煤炭消费总量控制。加大工地扬尘和道路扬尘管控，强化“禁烧区”露天烧烤管控，严格落实秸秆禁烧责任制，确保空气质量持续改善。

坚持不懈治水。全面推进“水十条”任务落实，突出抓好老旧城区雨污分流改造，持续做好建成区黑臭水体整治，加快小清河污染治理，确保年底前实现小清河辛丰庄出境断面水质稳定达标。实施小清河生态景观带改造提升工程。扎实开展农村饮水安全两年攻坚行动，突出抓好饮用水水源地环境问题整治。高起点规划建设白云湖、济西、白泉等湿地公园，科学实施湿地保护与修复工程，打造城市“绿肾”和市民休闲胜地。落实《济南市名泉保护条例》，持续开展泉水资源动态监测，科学利用地下水，确保泉群持续喷涌。

坚持不懈治山。按照“共抓大保护、不搞大开发”的原则，进一步实施生态补偿机制，持续推进南部山区自然生态景观保护。认真落实《济南市山体保护办法》，加大城区内158座山体的保护力度，强力推进破损山体治理，绿化提升山体27座，新开工建设郊野公园6处，完成新造林10万亩，推进实施古树名木保护工程，让广大市民的“幸福账单”上增加更多的“绿色收入”。

坚持不懈治土。认真落实“土十条”和土壤污染防治责任制。加强涉重金属行业污染防控和危险废物管理，实施土壤污染治理和修复试点项目。强化畜禽养殖环境监管，减少化肥农药使用量。推进居民小区和公共区域垃圾分类试点，坚决守住土壤环境质量底线，让土地变得更清洁干净。

（九）聚焦保障改善民生，塑造省会和谐稳定新优势。牢固树立以人民为中心的发展思想，围绕“七有”目标，着力解决结构性民生问题、实施普惠性民生工程、办好20件民生实事，坚决守住社会民生、安全稳定底线，打造一座有温度的城市。

千方百计扩大就业。把稳就业摆在突出位置，实施就业优先战略，落实积极就业政策，新增城镇就业12万人，确保“零就业”家庭动态清零。加大创业担保贷款支持力度，创业担保贷款额度从10万元提高到15万元以上。认真完成好军转干部、退役士兵安置任务，建立健全退役军人服务保障体系。实施高校毕业生就业促进计划，加强农民工服务中心和零工市场功能提升。做好城镇就业困难人员和登记失业人员等帮扶工作，努力实现弱有所扶、业有所就。

织密扎牢社保网络。坚持应保尽保、托住底线，巩固全民参保计划成果，提高城乡居民基础养老金，提高职工医保和居民医保待遇水平，建立失业保险金标准与最低工资标准挂钩联动机制，提高城乡低保和特困人员救助供养标准，全面推行“电子社保卡”。实施养老服务设施建设三年行动计划，加快市救助管理站改造、市社会福利院新建项目，新建区县养老中心3处、街道社区和农村养老设施160处。大力发展住房租赁市场，培育发展专业化规模化住房租赁企业，多渠道增加租赁住房供给，为符合条件的外来务工人员、新就业职工等提供租赁住房补贴，让他们感受到城市的关爱和温度。

优先发展教育事业。加快实施第三期学前教育三年行动计划和中心城区基础教育设施三年建设规划，开工建设标准化中小学、幼儿园140所，新增学位13万个。对区县政府履行教育职责开展评价，推行“优质园+”办园模式，推进学前教育普惠发展。推进“义务教育优质均衡发展区县”创建，促进普通高中多样化特色化发展。推动职业教育、民族教育、特殊教育、成人教育、民办教育等健康协调发展。持续抓好师德师风建设，实施校长综合能力提升工程，用教育家校长和高素质教师打造泉城教育品牌，撑起千百万家庭的未来和城市的未来。

持续建设健康济南。围绕建设国际医疗康养名城，加快建设国际医学科学中心，打造医教研融合发展的医学科学高地。加快健康城市、健康村镇建设试点和全省医养结合示范先行市建设，做好国家卫生城市复审工作。加强基层医疗卫生机构标准化建设，支持社会办医，积极开展“互联网+医疗健康”便民服务，推动电子社保卡、电子健康卡“两码合一”，加快推广电子病历，建立疫苗追溯体系。深入挖掘中医药技术，申办第六届中医科学大会，打造“扁鹊故里”品牌。按照“四个最严”的要求，聚焦人民群众关注的婴幼儿食品、网络配餐、互联网制售假药等重点领域，加大整治力度。广泛开展群众性体育活动，推动有条件的学校向社会免费开放体育设施和场馆，办好2019短池游泳世界杯、第四届国际持杖登山大会等赛事，让城市充满朝气、充满活力。

繁荣发展文化事业。高水平举办第八届山东文博会，组织开展“齐风鲁韵”系列演艺活动，办好全国非遗曲艺周，打造全国曲艺演出交流中心。加快规划建设新的市博物馆、科技馆、规划展览馆、档案馆、方志馆、少年宫、国家方志馆冶炼分馆，高起点规划建设非遗展示中心，加快遗产保护立法，推进“泉·城文化景观”申遗，实施文物拯救保护行动。深入开展“扫黄打非”工作，加强网络监管，确保文化市场稳定有序。深入挖掘各类传统文化特色资源，弘扬“济南战役”“莱芜战役”精神，传承好红色基因，开展庆祝建国70周年系列活动，让爱国融入血脉，让信仰激发力量。

优化创新社会治理。树立城市安全观，强化安全生产，实施“雪亮工程”，完善立体化社会治安防控体系，持续开展扫黑除恶专项斗争，保障人民群众生命财产安全，打造“平安济南”，决不让好人受气、坏人神气。推进城乡社区治理创新，推行“全科社区工作者”服务模式，发挥红白理事会、“泉城·爱帮”等阵地作用，广泛开展移风易俗实践活动。扎实开展双拥共建活动，深度推进军民融合发展，大力支持国防和军队建设改革。做好12345市民服务热线、信访、人民调解、仲裁和“七五”普法等工作，支持工青妇等群团组织面向基层一线参与社会治理，努力构筑共建共治共享的社会治理格局。

五、全面建设人民满意的行动政府

最强的力量就是说到做到，最好的落实就是一抓到底。今年是省委省政府确定的“工作落实年”，我们将顺应人民群众期待、适应现代化国际大都市建设要求，坚持只为成功想办法、不为困难找理由，牢固树立整体政府意识，打基础、立规章、抓落实、强督察，推动各项工作落地落细落实。

（一）以高度的自觉强党建。牢固树立“抓好党建是最大政绩”的理念，在加强政府系统党的建设上狠下功夫。坚持用习近平新时代中国特色社会主义思想武装头脑，增强“四个意识”，坚定“四个自信”，坚决维护习近平总书记在党中央的核心、全党的核心地位，坚决维护党中央权威和集中统一领导。深入推进“两学一做”学习教育常态化制度化，巩固提升“三照三转三改三推”活动成果，精心组织开展“不忘初心、牢记使命”主题教育，自觉在推动发展、服务人民中提升工作水平，把忠诚和看齐写在岗位上、刻在事业中。

（二）以法治的思维履职责。全面落实法治政府建设实施纲要，做到“法无授权不可为、法定职责必须为”，加快建设“法治济南”。依法接受市人大及其常委会法律监督和工作监督，自觉接受市政协民主监督，认真听取各民主党派、工商联、无党派人士和人民团体意见，认真办理人大代表议案建议和政协提案，完善行政执法和监督体系，推行重大行政决策民主公开。按照“做过的有规范，没做过的有预案”的原则，加快构建系统完备、科学规范、运行高效的依法行政制度体系，鼓励各级各部门争创国家级社会管理和公共服务综合标准化示范单位，切实做到事事有标准可依、岗位有标准规范、人人按标准履职。

（三）以一流的境界谋发展。教育引导政府系统的广大党员干部，树立攀强进位的雄心壮志，善于跳出济南看济南、站在全省看济南、放眼全国看济南、走向世界看济南，全面开展寻标对标活动，敢于和最好的比、与最强的赛，在更高的参照系中确定发展坐标和定位，从思想到工作、从思路到举措、从推进到成果，横向比较争先进、纵向比较促跨越，勇于争先率先领先，努力实现更好质量、更高效益、更快速度的发展，当好全省走在前列的排头兵。

（四）以变革的精神抓落实。切实强化创新意识，深入落实容错纠错机制，激励干部勇于担当，让政府系统的党员干部从“惯例”中走出来，从“框框”中跳出来，在吃透上级精神的基础上，紧密结合济南实际，创造性抓好贯彻落实。切实强化问题意识，按照“走到听到看到落实到”的原则，增强发现问题的敏锐、正视问题的清醒、解决问题的自觉，凡是群众反映强烈的问题都要严肃认真对待，凡是损害群众利益的行为都要坚决纠正。切实强化执行意识，实行工作项目化、项目目标化、目标节点化、节点责任化，建立推动高质量发展的指标体系和标准体系，强化干部绩效评价和政绩考核，做到凡事有人干、凡事有人管、事中事后有监督。

（五）以严实的作风促廉政。坚定不移推进全面从严治党，严格落实主体责任和“一岗双责”，深入推动全面从严治党走向严紧硬。认真落实中央八项规定及实施细则精神，持续纠正“四风”，坚决破除形式主义、官僚主义。始终保持惩治腐败“零容忍”高压态势，从严查处侵害群众利益的行为，既要干事又要干净，坚决守好廉洁底线。加强重点领域和关键环节审计，加强公共资金、国有资产、国有资源、政府项目、公共资源交易等廉政风险点防控。深入推进政务公开，始终把政府工作放在全社会的“聚光灯”下，提高政府的公信力。

各位代表，时势造英雄，实干开新篇！让我们更加紧密地团结在以习近平同志为核心的党中央周围，在市委的坚强领导下，只争朝夕、锐意进取，雷厉风行、快干实干，为建设“大强美富通”现代化国际大都市而努力奋斗！
